# Genetic predisposition to chronic lymphocytic leukemia

**DOI:** 10.1097/HS9.0000000000000194

**Published:** 2019-06-30

**Authors:** Philip J. Law, Richard S. Houlston

**Affiliations:** Division of Genetics and Epidemiology, Institute of Cancer Research, Sutton, Surrey, United Kingdom

**Take home messages**

- Chronic lymphocytic leukemia (CLL) is characterized by having one of the strongest familial risks of any cancer.
- Genome-wide association studies have identified common variants mapping to over 40 regions of genome that influence the risk of developing sporadic CLL. Sequencing of familial CLL has implicated rare loss-of-function mutations in shelterin complex genes in CLL predisposition.
- As well as providing new insights in the developmental basis of CLL, the cancer gene discovery initiatives have potential to inform the development of new therapeutic agents.

## Introduction

Chronic lymphocytic leukemia (CLL) is an indolent B-cell malignancy that has a strong inherited component, as evidenced by the 8-fold increased risk seen in relatives of CLL patients.^1▪^ Until recently, inherited genetic basis to CLL was unknown. Our understanding of CLL genetics has been transformed by the genome-wide association studies (GWAS) of CLL performed over the last 10 years.^2▪^^,^^3^^,^^4▪^^,^^5^ These GWAS have provided the first direct evidence for inherited susceptibility to CLL identifying common variants at over 40 independent genomic regions influencing risk of sporadic disease. In addition to common genetic variation influencing risk, high-throughput sequencing studies of CLL families have established a key role for rare disruptive mutations as determinants of disease susceptibility. Besides providing evidence for genetic susceptibility to CLL, the genetic regions and genes risk identified by these analyses have provided fresh insights into the biological basis of CLL development.

## Current state of the art

GWAS have so far identified single nucleotide polymorphisms (SNPs) at 43 independent genetic regions that influence the risk of developing sporadic CLL.^2▪^^,^^3^^,^^4▪^^,^^5^ While the risk of CLL associated with each of the GWAS risk SNPs is modest, in concert they have the potential to have more profound effects on an individual's risk of developing CLL. Thus far the currently identified risk SNPs account for 25% of the heritable risk. By fitting all SNPs from GWAS simultaneously using statistical modeling has shown that the estimated heritability of CLL attributable to all common variation is 34%, thus having potential to explain 57% of the overall familial risk^4▪^ and confirming the long held belief that a significant part of the heritable risk of CLL is polygenic in nature.^6^

**Figure 1:**
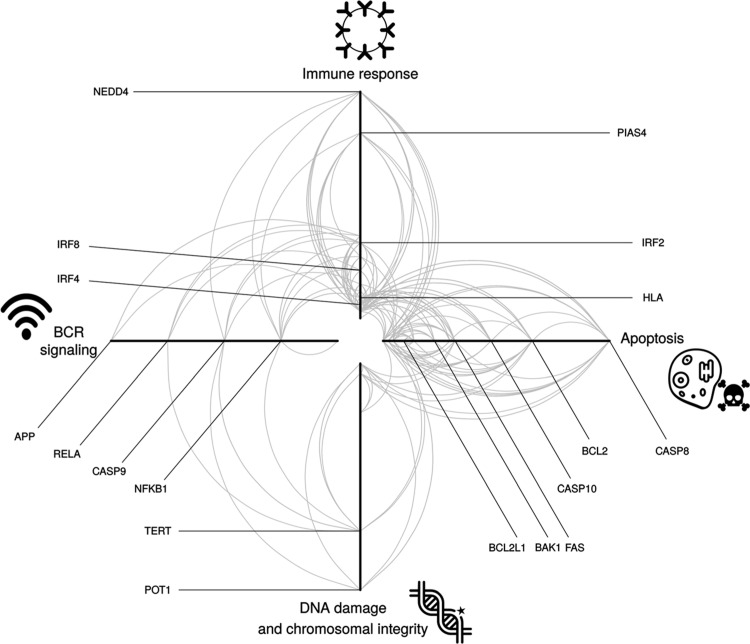
**Genes implicated by genome-wide association studies as having a role in defining the development of CLL.** Each arm corresponds to a defined biological process. Each arc represents an interaction between 2 proteins, and the distance from the center of the plot corresponds to a greater number of protein-protein interactions (higher degree of the node). Selected proteins known to be involved in CLL risk are indicated. CLL = chronic lymphocytic leukemia.

To the extent that they have been deciphered, most cancer GWAS risk regions map to noncoding regions of the genome and influence disease risk by altering gene regulation.^7▪^ This is also the case for CLL with over 75% of risk regions showing enrichment of active promoters and/or enhancers when assessed by H3K27ac, H3K4me3, and H3K4me1 ChIP-seq marks. Moreover, CLL risk regions are enriched for CLL-related regulatory elements which are CLL-specific or show differential regulation across CLL and B-cell development.^4▪^ Quantitative trait locus analysis in conjunction with data from H3K27ac, ATAC-seq, and DNA methylation profiling are consistent with risk regions mediating their effects by influencing chromatin activity. The identified loci show an over-representation of transcription factor (TF) binding. Several of the TFs mapping to risk regions have well-established roles in B-cell function; for example, OCT2, IRF4, and RUNX3 being targeted for hypomethylation in B-cells. MYC is a direct target of IRF4 in activated B cells, with IRF4 itself being a direct target of MYC transactivation. In this respect, it is noteworthy that genetic variants at IRF4 and MYC are recognized factors for CLL pathogenesis. Collectively, findings from these analyses are consistent with CLL GWAS risk SNPs mapping within regions of active chromatin state that exert effects on B-cell cis-regulatory networks. Investigating the genetic pathways between the gene products in proximity to the GWAS SNPs, it has been shown that gene products are primarily involved in immune response, B-cell receptor (BCR)-mediated signaling, apoptosis, and maintenance of chromosome integrity, as well as interconnectivity between the gene products all being central to B-cell development (Fig. 1). It is notable that Ibrutinib (a BTK inhibitor) and Idelalisib (a PI3KCD inhibitor) mediate their effects through interference of BCR signaling, and Venetoclax targets the antiapoptotic behavior of BCL-2.

Families segregating CLL have provided evidence for Mendelian susceptibility; however, until recently, the identification of rare alleles with large effects has been elusive. The identification of this class of susceptibility is especially important because mutations are causal and provide direct insight to cancer biology, in contrast to GWAS associations. By performing whole-exome sequencing of CLL families, loss-of-function mutations in Protection of Telomeres 1 (POT1) and other components of the shelterin complex have been demonstrated.^8▪^^,^^9^ As well as providing support for the role of rare variants these findings further highlight telomere dysregulation as a key process in CLL development. Moreover, they extend the spectrum of cancer associated with inherited mutations in these genes. It is, however, likely that shelterin complex gene mutations confer cancer risks analogous to those associated with ATM heterozygosity^10^ or CHEK2 for breast cancer.^11^ Nevertheless, because the dysregulation of telomere protection has been identified as a target for potential therapeutic intervention in CLL, it may be possible that early identification of mutation carriers will facilitate improvements in future disease management.

## Future perspectives

Recent studies have provided the first direct evidence for inherited predisposition to CLL. As well as providing new insights in the developmental basis of CLL, these gene discovery initiatives have the potential to impact on risk prediction and on the successful development of new therapeutic agents. Since much of the inherited risk of CLL still remains unexplained additional studies based on larger datasets offer an opportunity to identify new risk regions and susceptibility genes. Deciphering the function of GWAS risk loci is an important step toward testable hypotheses regarding the biological processes involved in pathogenesis. Elucidating the mechanisms through which noncoding variants exert their effect is, however, challenging as the genotyped SNP is not generally a strong candidate for causality. While studies to fully elucidate the regulatory mechanisms underpinning risk regions are in their relative infancy, such endeavors are likely to involve high-throughput systems such as massive parallel reporter assays and exploration of tissue-specific effects in appropriate model systems and CRISPR-Cas9-mediated disruption of candidate regulatory elements.
